# Phylogenetic diversity of microorganisms in subseafloor crustal fluids from Holes 1025C and 1026B along the Juan de Fuca Ridge flank

**DOI:** 10.3389/fmicb.2014.00119

**Published:** 2014-03-25

**Authors:** Sean P. Jungbluth, Huei-Ting Lin, James P. Cowen, Brian T. Glazer, Michael S. Rappé

**Affiliations:** ^1^Hawaii Institute of Marine Biology, University of Hawaii at ManoaKaneohe, HI, USA; ^2^Department of Oceanography, University of Hawaii at ManoaHonolulu, HI, USA

**Keywords:** deep subsurface, microorganisms, diversity, Juan de Fuca Ridge, SSU ribosomal RNA gene, Ocean Drilling Program

## Abstract

To expand investigations into the phylogenetic diversity of microorganisms inhabiting the subseafloor biosphere, basalt-hosted crustal fluids were sampled from Circulation Obviation Retrofit Kits (CORKs) affixed to Holes 1025C and 1026B along the Juan de Fuca Ridge (JdFR) flank using a clean fluid pumping system. These boreholes penetrate the crustal aquifer of young ocean crust (1.24 and 3.51 million years old, respectively), but differ with respect to borehole depth and temperature at the sediment-basement interface (147 m and 39°C vs. 295 m and 64°C, respectively). Cloning and sequencing of PCR-amplified small subunit ribosomal RNA genes revealed that fluids retrieved from Hole 1025C were dominated by relatives of the genus *Desulfobulbus* of the *Deltaproteobacteria* (56% of clones) and *Candidatus* Desulforudis of the *Firmicutes* (17%). Fluids sampled from Hole 1026B also contained plausible deep subseafloor inhabitants amongst the most abundant clone lineages; however, both geochemical analysis and microbial community structure reveal the borehole to be compromised by bottom seawater intrusion. Regardless, this study provides independent support for previous observations seeking to identify phylogenetic groups of microorganisms common to the deep ocean crustal biosphere, and extends previous observations by identifying additional lineages that may be prevalent in this unique environment.

## Introduction

Several studies now support the notion that the enormous volume of sediments and basement basalt that compose the global system of mid-ocean ridge spreading centers, flank and ocean basins harbors microbial life (e.g., Gold, 1992; Parkes et al., 1994; Fisk et al., 1998; Bach and Edwards, 2003; Cowen, 2004; D'Hondt et al., 2004; Schrenk et al., 2010; Orcutt et al., 2011a; Jungbluth et al., 2013). Fueled by cooling of basement rock, fluid circulation occurring within porous and permeable young ridge flanks (<10 million years) introduces terminal electron acceptors into the ocean crust, making the uppermost igneous basement a suitable marine subsurface environment for microorganisms (Baross et al., 2004). Although the uppermost (40–500 m) basement aquifer is estimated to contain ~2% of the world ocean volume (Johnson and Pruis, 2003), information regarding the evolutionary history, community structure, functional properties, and metabolic activity of microorganisms inhabiting this unique system is scarce (Schrenk et al., 2010; Orcutt et al., 2011a).

Most mid-ocean ridge flank and ocean basin basement is buried under thick, impermeable layers of sediment that significantly restrict sampling opportunities. However, Circulation Obviation Retrofit Kit (CORK) observatories (Davis et al., 1992; Edwards et al., 2011) affixed to Ocean Drilling Program (ODP) and Integrated Ocean Drilling Program (IODP) boreholes (Davis and Becker, 2001; Fisher et al., 2005) offer access to perform measurements and experiments *in situ* or collect crustal fluids. Fluids within the basement rock can be channeled up through the sediment horizon via fluid delivery lines and collected from sampling ports at the seafloor via submersible (Cowen et al., 2003; Huber et al., 2006; Cowen et al., 2012; Edwards et al., 2012; Lin et al., 2012; Nigro et al., 2012; Jungbluth et al., 2013).

**Figure 1:**
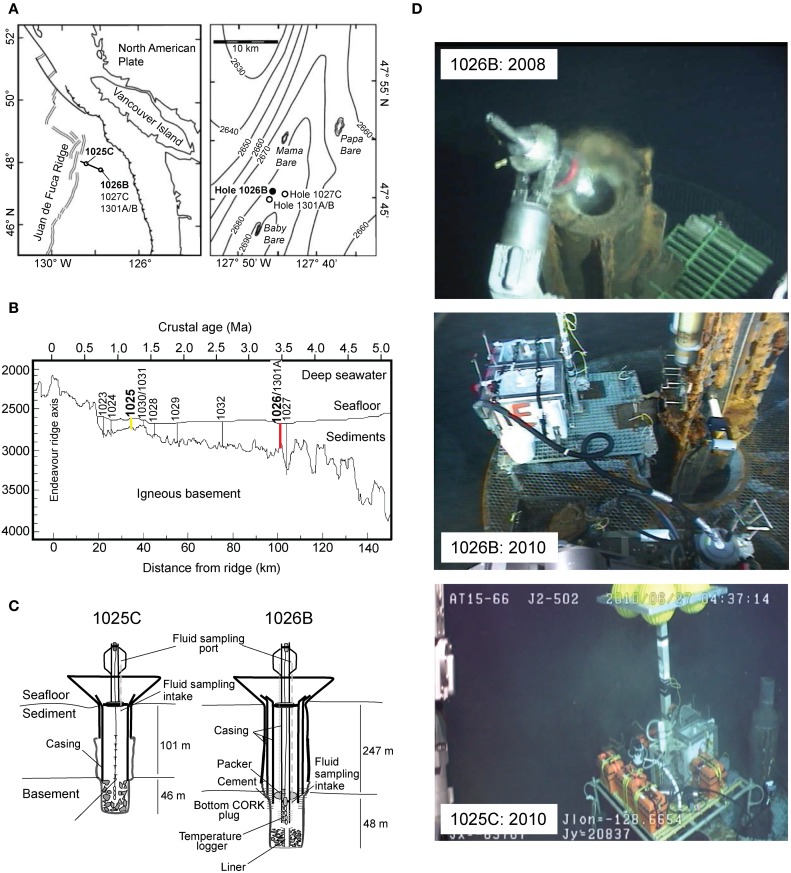
**(A)** Location of CORK observatory sampling sites on the Juan de Fuca Ridge flank, Pacific Ocean. **(B)** Cross-sectional diagram of ODP Leg 168 showing depth of basement crust and sediment thickness, basement age and associated distance from ridge axis, and locations of Holes 1025 (yellow) and 1026 (red) in boldface font (modified from Wheat et al., 2004). **(C)** Schematic diagram of CORKs located at Holes 1025C and 1026B (not drawn to scale). Fluids were sampled from the exit valve of the fluid delivery line (modified from Lin et al., 2012). **(D)** Photo of squeeze sample taken for biogeochemical analysis from top of CORK 1026B in 2008, fluid sampling device used in 2010 at Hole 1026B, and Borehole Flushing Unit and fluid sampling device used at Hole 1025C in 2010.

During ODP Leg 168, an array of boreholes were drilled into ocean basement of increasing age along a transect perpendicular to the Juan de Fuca Ridge (JdFR) axis on its eastern flank (Figures 1A,B) (Shipboard Scientific Party, 1997). Two of these, ODP Holes 1025C and 1026B, penetrate over-pressured basaltic crust and were sealed with CORK sampling platforms. The sediment cover at Hole 1026B is sufficiently thick to act as an impermeable seal (Embley et al., 1983), preventing circulating basement fluids from directly mixing with deep ocean seawater, while Hole 1025C lies within a transition zone between sediment-free regions that may allow for open hydrothermal circulation and sediment-covered, hydrologically sealed igneous crust (Shipboard Scientific Party, 1997). While both boreholes were originally equipped with early-generation CORKs that delivered crustal fluids directly through a potentially reactive iron casing (Davis et al., 1992; Shipboard Scientific Party, 1997), in 2004 the CORK at Hole 1026B was replaced with an upgraded CORK-II, which is more amenable to microbiological sampling due to dedicated stainless steel fluid delivery lines that circumvent fluid passage through the casing itself (Becker and Davis, 2005). Also in 2004, Hole U1301A was drilled in close proximity to Hole 1026B and affixed with a CORK-II and stainless steel fluid delivery lines (Expedition 301 Scientists, 2005).

Several studies have used the CORK observatories along the JdFR flank to investigate the coupled microbiology and chemistry of basalt-hosted crustal fluids in this region (Cowen et al., 2003; Huber et al., 2006; Nakagawa et al., 2006; Steinsbu et al., 2010; Orcutt et al., 2011b; Jungbluth et al., 2013). From these and other studies (e.g., Wheat et al., 2004; Lin et al., 2012) it is now known that basaltic crustal fluids are enriched in several compounds that are highly likely to impact biological processes in this system, including methane, hydrogen, ammonium, and iron, and are depleted in others, including magnesium, phosphate, nitrate, sulfate, and dissolved organic carbon (DOC), relative to bottom seawater. In addition to mineral weathering and serpentinization, the chemical composition of these fluids also suggests that microbially-mediated processes including biogenic methane cycling, iron metabolism, sulfate reduction and fermentation could also be occurring, with microorganisms drawing down DOC, nitrate, phosphate, and sulfate stocks in the process. Consistent with some of these processes, several microbial lineages identified from Holes 1026B or U1301A fluid samples (Cowen et al., 2003; Huber et al., 2006; Jungbluth et al., 2013) and solid substrates (Nakagawa et al., 2006; Steinsbu et al., 2010; Orcutt et al., 2011b; Lever et al., 2013) via both culture-based and cultivation independent studies are related to Bacteria and Archaea known to transform sulfur compounds, including the bacterial lineages Candidatus *Desulforudis, Desulfocapsa, Thiomicrospira, Sulfurimonas*, and the archaeal lineage *Archaeoglobus*. Some of these may grow chemolithoautotrophically via the coupling of hydrogen oxidation to sulfate reduction. However, many of the microbial lineages identified via phylogenetic marker-based cultivation independent methods within basalt-hosted deep subsurface fluids of the JdFR flank are not closely related to any known cultivated strains, and possess unknown functional roles in this system (Jungbluth et al., 2013).

In this study, we sought to use a phylogenetic marker-based approach in order to compare the phylogenetic identity of microorganisms inhabiting fluid samples retrieved from three JdFR flank CORK observatories sampled within the span of 1 week: 1025C, 1026B, and 1301A, and to compare these samples to similar data derived previously from fluids retrieved from Hole 1026B. Our goals were to understand how CORK characteristics may influence both the microbial community structure and underlying chemistry of fluid samples from CORKS in close proximity but of different design and construction, as well as to understand how changes to the CORK at a single borehole (1026B), and the loss of CORK integrity at this location over time, impact the resulting chemical and microbial properties of the resultant fluids.

## Materials and methods

### Sample collection

During R/V *Atlantis* cruise AT15-66 (13 June–1 July 2010), fluids were collected from CORK observatories at ODP Holes 1025C (47°53.247'N, 128°38.919'W) and 1026B (47°45.759'N, 127°45.552'W), located 34 and 101 km east of the JdFR spreading center, respectively (Figures 1A,B). Fluids were sampled from the microbiological and geochemical sampling lines associated with the original 1025C CORK (Davis et al., 1992) and 1026B CORK-II (Fisher et al., 2005) installations. Holes 1025C and 1026B penetrate 101 and 247 m of sediments and another 46 and 48 m into ~1.2 and ~3.5 Ma basement rock, respectively (Table 1; Figure 1C). In 2008, a small volume (~150 mL) fluid sample was collected from Hole 1026B on HOV *Alvin* dive 4432 during R/V *Atlantis* cruise AT15–35 using a squeeze sampler while the CORK head was opened for redeployment of downhole instrument strings (Figure 1D); the volume of this sample was only sufficient for geochemical analysis.

**Table 1:** **Characteristics of fluid samples collected from Holes 1025C, 1026B, U1301A, and background bottom seawater**.

	**1025C^a^**	**1026B^a^**	**U1301A^b^**	**Bottom seawater^b^**
Clone prefix	1025C10	1026B10	1301AXX^c^	1301AXXsw^c^
Sampling depth (m)	2606 + 101 + 46^d^	2658 + 247 + 48^d^	2667 + 262 + 108^d^	
Sampling temp (°C)	15	4	18	n.d.
pH	7.9	6.8 (7.6)^e^	7.4	7.7
O_2_ (μM)	18	88	10	80
Ca^2+^ (mM)	30.4 (34.1)	12.0 (55.8)^e^	53.0	10.4
Mg^2+^ (mM)	29.7 (25.2)	48.5 (2.7)^e^	3.40	53.7
K^+^ (mM)	9.4 (9.2)	9.6 (6.5)^e^	6.4	10.2
CH_4_ (μM)	5.3 (6.3)	n.d. (2.0)^b^	1.5	0.0002^f^
H_2_ (μM)	n.d. (n.d.)	n.d. (0.7)^b^	0.3–2	0.0004^f^
NH^+^_4_ (μM)	43 (51)	2.5 (122)^e^	100	<0.05
PO^3-^_4_ (μM)	0.10 (0)	<0.01 (0.9)^g^	0.14	2.89
NO^−1^_2_+ NO^2-^_3_ (μM)	6.4 (0)	40.6 (0)	1.5	40.8
SO^2-^_4_ (mM)	26.2 (25.9)	27.2 (17.7)^e^	18.3	28.4
Fe^2+^ (μM)	1.23 (1.46)	1000	1.5	<0.1
Dissolved Fe (μM)	1.39 (1.65)	1130 (1.1)^g^	3.15	<0.1
Total Fe (μM)	39.07 (46.4)	1360	3.52	<0.1
DOC (μM)	22 (19)	43 (8–17)^h^	12	39
Alkalinity (meq/L)	0.88 (0.58)	0.36 (0.53)^e^	0.52	2.48

All chemical data was collected in 2010 unless otherwise noted.

a For some parameters, corrected values representing end-member fluids (nitrate = 0 μM) appear in parentheses.

b Geochemical data from Lin et al. (2012).

c Jungbluth et al. (2013).

d Water column depth at CORK sampling spigot + sediment + permeated basement rock.

e Cowen et al. (2003).

f Kelley et al. (1998).

g Wheat et al. (2004).

h Lang et al. (2006).

n.d., not determined.

In 2010, a deep-sea pumping system incorporating a non-contaminating titanium and Teflon pump head and complementary in-line sensors was used to draw and collect fluids from Holes 1025C and 1026B into custom-made 60-L acid-washed Tedlar bags (MiDan Co., Chino, CA, USA) protected by a high-density polyethylene box (Large Volume Bag Sampler, LVBS, Cowen et al., 2012; Lin et al., 2012; Jungbluth et al., 2013) (Figure 1D). The fluid flowpath contained a custom flowcell with an *in situ* oxygen optode (Aanderaa Data Systems, Bergen, Norway), temperature sensor (Sea-Bird Electronics Inc., Bellingham, WA, USA), and a custom fluid flow sensor to allow for real-time assessment of the integrity of fluid connections and plumbing flowpath (e.g., Cowen et al., 2012). All tubing and sampling bags along the fluid delivery path were acid washed prior to deployment on the submersible. Borehole fluids were flushed through the CORK observatory fluid delivery lines prior to sampling into the LVBS. Hole 1025C was flushed overnight using a Borehole Flushing Unit pump to clear out the section of 10.75” iron casing while less time (~30 min) was used to flush Hole 1026B due to the much smaller void volume of the 1/4” fluid delivery line (Figure 1D). Samples for microbiological analysis were collected on ROV *Jason II* dives J2–502 (1025C) and J2–500 (1026B).

Subsamples for geochemistry were taken immediately after shipboard retrieval of the LVBS samplers by transferring fluids directly to gastight syringes (Hamilton Co., Reno, NV, USA) or via acid-washed tubing and a peristaltic pump into acid-cleaned high-density polyethylene (HDPE) bottles. All geochemical sub-sampling was conducted inside a laminar flow hood with a high-performance air filter (HEPA) to ensure a clean sampling environment. Samples for major ion analysis were stored refrigerated, while samples for inorganic nutrients (nitrate, nitrite and phosphate), DOC and total organic carbon (TOC) were stored frozen until further processing. Subsequently, 1.95 L (1025C) and 1.50 L (Hole 1026B) of sample fluids were passed through 0.22 μm pore-sized Sterivex-GP filter cartridges (Millipore Corporation, Billerica, MA, USA) for the collection of microbial biomass. Filters were subsequently stored in 2.0 ml of DNA lysis buffer [20 mM Tris-HCl, 2 mM EDTA, 1.2% Triton X-100, 2% lysozyme (w/v), pH 8] at −80°C until further processing. All samples were stored frozen within 2 h of shipboard retrieval of the bag samplers.

As described previously, seawater samples for comparison were collected in the vicinity of Hole 1026B on cruises in 2008, 2009, and 2010 (Lin et al., 2012; Jungbluth et al., 2013).

### Genomic DNA extraction and rRNA gene sequencing

In a shore-based laboratory, Sterivex membrane filters were subsequently thawed to room temperature and extracted for DNA using the PowerSoil DNA isolation kit (MO BIO Laboratories, Carlsbad, CA, USA) following the manufacturer's protocol. Quantification of the resulting genomic DNA was performed using a NanoDrop DN-100 spectrophotometer (Thermo Fisher Scientific, Waltham, MA, USA). Small subunit ribosomal RNA (SSU rRNA) gene fragments were amplified via the polymerase chain reaction (PCR) using the universal oligonucleotide forward and reverse primers 519F (5'-CAGCMGCCGCGGTAATWC-3') and 1406R (5'-ACGGGCGGTGTGTRC-3'), respectively (Lane et al., 1985). Each 20 μl PCR reaction contained 0.25 U of PicoMaxx high fidelity DNA polymerase (Stratagene, La Jolla, CA, USA), 1x PicoMaxx reaction buffer, 200 μM of each deoxynucleoside triphosphate (dNTPs), 200 nM of both forward and reverse primer, and ~4–8 ng of environment DNA template. Appropriate positive and negative control reactions were also included. PCR cycling conditions consisted of a denaturation step at 95°C for 4 min, followed by 30 cycles of 95°C denaturation for 30 s, 55°C annealing for 1 min, 72°C extension for 2 min, and a final extension step at 72°C for 20 min. Amplification products were size-selected and cleaned using the QIAquick gel extraction kit (Qiagen, Valencia, CA, USA) and subsequently cloned with the TOPO TA Cloning kit (Invitrogen, Carlsbad, CA, USA) following the manufacturer's instructions. Clones were sequenced using an ABI 3730XL DNA Analyzer (Applied Biosystems, Carlsbad, CA, USA).

### Phylogenetic analysis

DNA sequences were trimmed of vector sequence and manually curated using Sequencher version 4.9 software (GeneCodes, Ann Arbor, MI, USA) and subsequently checked for chimera formation via Bellerophon (Huber et al., 2004) and CHECK_CHIMERA, available from the Ribosomal Database Project (Cole et al., 2005). Using the ARB software package (Ludwig et al., 2004), hand-curated clone sequences were aligned with version SSURef_111 of the SILVA ARB database (Pruesse et al., 2007) modified to include short (<1200 nucleotides) environmental gene clones that were highly similar to clone sequences obtained in this study. Phylogenetic analyses were performed with the RAxML maximum likelihood method using the general time-reversible model with a gamma distributed rate variation for nucleotide substitution (Stamatakis, 2006) and selection of the tree with the highest likelihood value based on 100 simulations. Sequences of short length were added to the maximum-likelihood-derived phylogenies using the parsimony insertion tool in ARB. Bootstrap analyses employed RAxML (Stamatakis et al., 2008) via the CIPRES Portal V 3.1 (Miller et al., 2010). All non-redundant sequences generated in this study have been deposited in GenBank under accession numbers KF574286-KF574384.

### Microbial community analysis

Microbial community α-diversity estimators, rarefaction curves, and community relatedness were generated or assessed using lane-masked (community relatedness) or unmasked (α-diversity estimators, rarefaction curves) clone sequences grouped into operational taxonomic units (OTUs) defined at 99 and 97% SSU rRNA gene sequence similarity cut-off values using the average neighbor clustering method as implemented by the mothur software package (Schloss et al., 2009). Microbial richness, evenness, and diversity were assessed by the Chao1 richness estimator (S_chao1_) (Chao, 1984), Simpson evenness index (E_simpson_) (Simpson, 1949), and the non-parametric Shannon diversity index (Ĥ_shannon_) (Shannon, 1948), respectively, as implemented in mothur (Schloss et al., 2009).

### Analytical methods for geochemistry

Subsamples for shipboard colorimetric measurement of iron species by UV-vis spectrophotometer using the ferrozine method (modified after Stookey, 1970) were split into three aliquots. For Fe(II), samples from a gastight syringe were 0.2-μm filtered in an N_2_ atmosphere and immediately reacted with a ferrozine solution consisting of 50% of a 2.5 M ammonium acetate buffer and 50% of 0.01 M ferrozine. Samples were allowed to stand for ~30 min for color development, followed by absorbance measurement at 562 nm. Total dissolved iron (Fe_d_) was derived by reacting a separate 0.2-μm filtered aliquot for 24 h with 0.2 M hydroxylamine in 0.1 M HCl prior to ferrozine analysis. Total iron (Fe_T_) was determined by reacting an unfiltered aliquot for 24 h with 0.2 M hydroxylamine in 0.1 M HCl prior to ferrozine analysis. Major ions were analyzed with a Dionex ICS-1100s ion chromatograph (Thermo Fisher Scientific, Sunnyvale, CA, USA). In addition, magnesium and calcium were analyzed shipboard with ethylene diamine tetraacetic acid (EDTA) and ethylene glycol tetraacetic acid (EGTA) titration (Grasshoff et al., 1999). Nitrate, nitrite and phosphate were analyzed by spectrophotometric methods (Grasshoff et al., 1999). Ammonium was measured by a modified flow-injection fluorescence method (Jones, 1991). Methane and hydrogen were measured by gas chromatography (Lin et al., 2012).

### End-member correction

Bottom seawater entrainment at the time of sampling (e.g., via leaks in sampling equipment or compromised integrity of the CORK seal) was estimated using a two end-member mixing model (Libes, 2009) based on nitrate concentration (Mottl et al., 1998; Wheat and Mottl, 2000). In anoxic basement fluids, our working assumption is that end-member nitrate concentration is zero because nitrate is exhausted (G. Wheat, personal communication), while bottom seawater contains ~40 μM nitrate in this region (Wheat et al., 2010; Lin et al., 2012).

## Results

### Biogeochemical characteristics of borehole fluid samples

Dissolved oxygen concentrations in fluids collected from CORK observatories immediately revealed that 1026B (O_2_ = 88 μ M) showed signs of bottom seawater intrusion, while fluids from 1025C and 1301A were deplete in O_2_ (18 μ M and 10 μ M, respectively) relative to bottom seawater (110 μ M). After end-member correction to a nitrate concentration of 0 μ M, the 6.4 μ M of nitrate measured in the Hole 1025C fluid sample suggests that it consisted of ~84% basement fluid and ~16% seawater (Table 1). The concentration of nitrate in the Hole 1026B fluid sample characterized here was nearly identical to that in bottom seawater (40.6 vs. 40.8 μ M), while the concentration of dissolved iron was far higher than that in a sample from Hole 1026B collected from the top of the open CORK in 2008 (1000 μ M vs. ~0.5 μ M, respectively). The lower magnesium and sulfate and higher calcium and ammonium relative to bottom seawater suggests that end-member basement fluid represented only ~3–10% of the 1026B fluid sample characterized here. It is not known at what time between 2008 and 2010 sampling periods that 1026B became catastrophically compromised to bottom seawater intrusion; however, end-member chemical concentrations calculated for crustal fluids accessed via 1026B in 2008 were 77% end-member basement fluid. Overall, the end-member basement fluid from Hole 1026B is more similar to that from Hole U1301A than Hole 1025C, consistent with previous observations (Elderfield et al., 1999; Wheat et al., 2004).

### Microbial community structure

A total of 734 and 754 ng of DNA was extracted from Holes 1025C and 1026B fluid samples, respectively. After amplification with universal oligonucleotide primers and cloning, 70 (1025C) and 87 (1026B) SSU rRNA gene clones were sequenced (Table 2). Microbial communities were analyzed using a variety of α-diversity calculators and OTUs defined at 99% and 97% SSU rRNA gene sequence similarity, resulting in 22 (99%) and 16 (97%) OTUs from 1025C and 53 (99%) and 42 (97%) OTUs from 1026B. The Shannon diversity index was depressed in the Hole 1025C sample, indicating that this sample possessed lower community diversity, while rarefaction curves and Chao1 richness estimators generated using the same OTU definitions indicated that the clone libraries were under sampled (data not shown).

**Table 2:** **Relative abundance of SSU rRNA gene clones from Holes 1025C and 1026B fluids**.

**Phylogenetic affiliation^a^**	**1025C (*n* = 70)**	**1026B (*n* = 87)**	**Representative clones**
***Archaea***			
* Crenarchaeota (thaumarchaeota)*			
Marine benthic group A	0	1	1026B_51
Marine group I	0	2	1026B_30, 1026B_73
* Euryarchaeota*			
DHVEG-6 (Figure 4D)	0	1	1026B_15
***Bacteria***			
* Bacteroidetes*			
* Marinilabiaceae*	1	0	1025C_63
NS9	0	2	1026B_18
SB-1	4	0	1025C_22, 1025C_61
* Cand.* phylum SAR406	3	3	1025C_18, 1026B_53, 1026B_13
* Chloroflexi*			
* Anaerolineaceae*	1	0	1025C_30
* Firmicutes*			
* Acholeplasmataceae*	1	0	1025C_27
* Clostridiaceae*			
* Alkaliphilus*	1	0	1025C_01
* Ca. Desulforudis* (Figure 4A)	17	0	1025C_25
* Peptococcaceae*			
* Desulfotomaculum*	1	0	1025C_05
RF3 (Figure 4B)	0	2	1026B_12
* Fusibacter*	0	1	1026B_60
* Planctomycetes*			
OM190	0	2	1026B_72
* Proteobacteria*			
* Alphaproteobacteria* (Figure 6)			
* Hyphomicrobiaceae*			
1301A10_076 lineage (Figure 6)	0	1	1026B_03
OCS116 (Figure 6)	0	1	1026B_24
* Rhodobacteraceae*			
* Roseobacter* clade NAC11-7 (Figure 6)	0	1	1026B_82
* Rhodospirillaceae*			
* Defluviicoccus*	0	1	1026B_23
SAR11	3	17	1025C_67, 1026B_29, 1026B_01, 1026B_20, 1026B_52, 1026B_80, 1026B_58, 1026B_41, 1026B_47, 1026B_87, 1026B_69
* Betaproteobacteria*			
* Burkholderiaceae*			
* Cupriavidus*	0	1	1026B_67
* Deltaproteobacteria* (Figure 3)			
* Desulfobacteraceae*			
* Desulfobacula*	0	6	1026B_07, 1026B_70
* Desulfococcus*	3	0	1025C_53
* Desulfobulbaceae*			
* Desulfobulbus* (Figure 3)	56	8	1025C_08, 1025C_15, 1026B_06, 1026B_21
* Desulfocapsa* (Figure 3)	1	0	1025C_51
1301A09_118 lineage	0	2	1026B_57
* Desulfohalobiaceae* (Figure 3)	0	5	1026B_19
* Desulfovibrionaceae* (Figure 3)	4	0	1025C_57
* Nannocystineae*	0	1	1026B_76
* Nitrospinaceae*	0	2	1026B_42
SAR324	0	1	1026B_55
* Epsilonproteobacteria*			
* Helicobacteraceae*			
* Sulfurimonas* (Figure 4C)	0	3	1026B_05, 1026B_25, 1026B_62
* Gammaproteobacteria* (Figure 5)			
9NBGBact_8 (Figure 5)	0	1	1026B_74
AGG47	0	2	1026B_34, 1026B_36
Arctic96BD-19	0	5	1026B_64, 1026B_46, 1026B_17
JTB35			
1301A10_105 lineage (Figure 5)	0	1	1026B_14
* Moraxellaceae*	1	0	1025C_31
OM182	0	1	1026B_83
* Pseudoalteromonadaceae*	0	9	1026B_66, 1026B_79, 1026B_77, 1026B_56, 1026B_35
* Pseudomonas*	0	5	1026B_40, 1026B_86
* Thiomicrospira* (Figure 5)	0	6	1026B_11
* Zetaproteobacteria*	0	1	1026B_31
* Verrucomicrobia*			
Arctic97B-4	0	1	1026B_59

a Phylogenetic affiliations were determined using SILVA SSU database release 111. In cases where the SILVA taxonomy was inconsistent, lineages were named after the first gene clone derived from the group.

**Figure 2:**
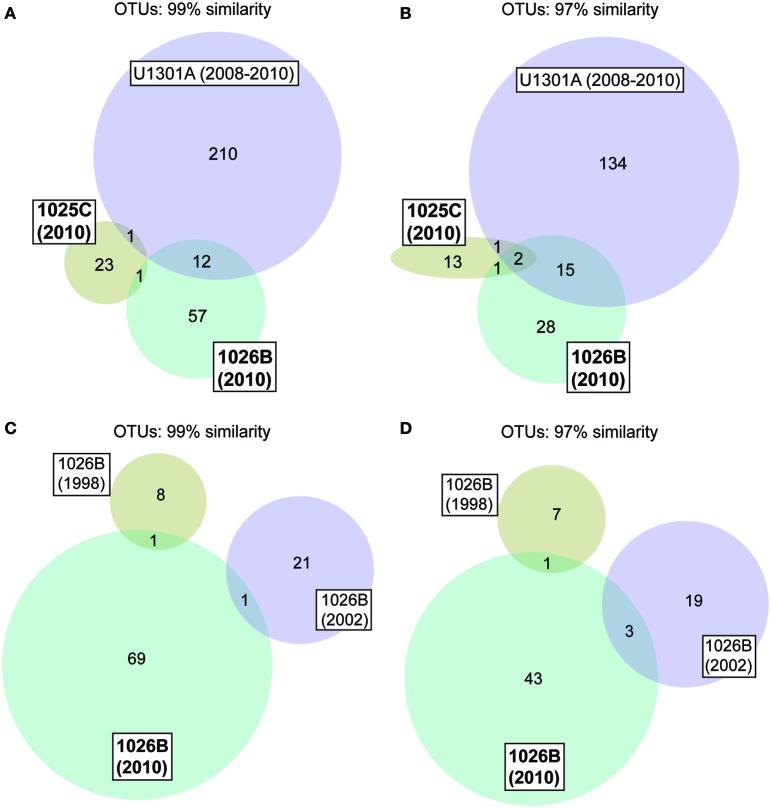
**Venn diagrams showing the overlap in microbial communities between Holes 1025C, 1026B, and U1301A fluids (A,B), and between Hole 1026B fluids collected in different years (C,D).** Data for “U1301A 2008–2010” appears in Jungbluth et al. (2013), “1026B 1998” in Cowen et al. (2003), and “1026B 2002” in Huber et al. (2006).

Phylogenetic analyses indicated that the borehole fluid samples described here contained a majority (~80%, 1025C) or significant fraction (~40%, 1026B) of clones related to microorganisms that harbor physiological attributes consistent with the physical and chemical conditions of life within the crustal subsurface environment (e.g., meso- and thermophiles, anaerobes, sulfate-reducers, etc.), or are related to SSU rRNA gene sequences previously recovered from related environments (Table 2). The 1025C and 1026B fluid samples characterized here showed little overlap in the taxonomic identity of OTUs, as well as little overlap with fluids sampled from Holes 1026B and U1301A previously (Figure 2) (Cowen et al., 2003; Huber et al., 2006).

### Hole 1025C fluid community structure

**Figure 3:**
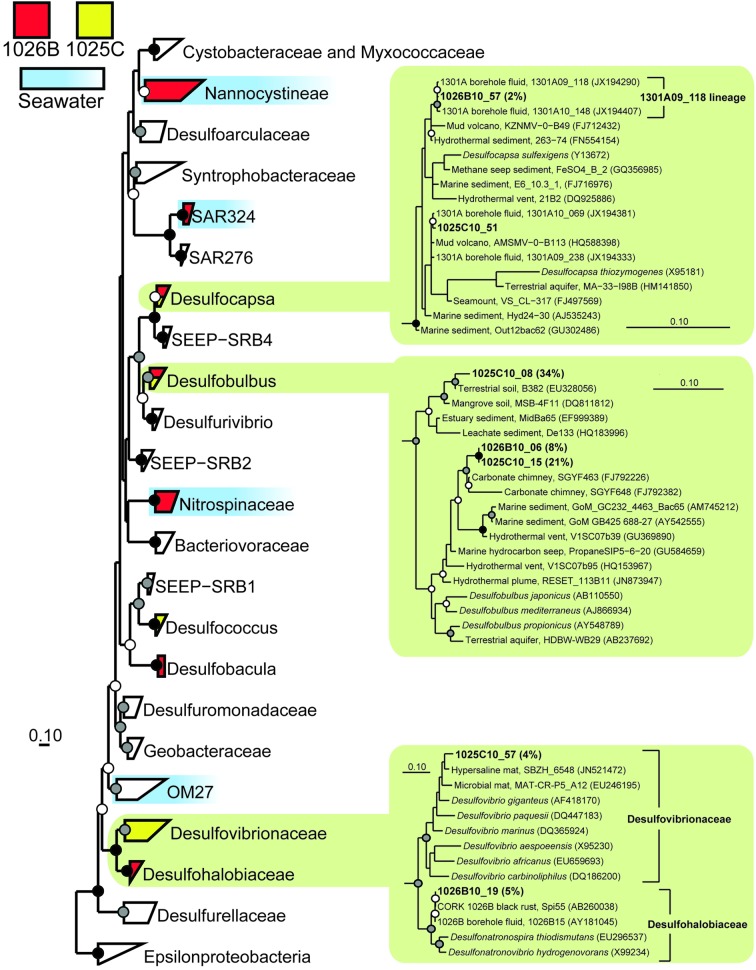
**Phylogenetic relationships of SSU rRNA gene clones related to the phylum *Deltaproteobacteria*, colored according to borehole of origin.** Clones derived from nearby bottom seawater are shown in blue (Jungbluth et al., 2013). Cultivated *Epsilonproteobacteria* were used as an outgroup (not shown). Detailed phylogenies are shown for selected lineages. Black (100%), gray (>80%), and white (>50%) circles indicate nodes with bootstrap support, from 1000 replicates. Gene clones recovered in this study are highlighted in bold font; the relative abundance of identical clones is listed in parentheses. The scale bars correspond to 0.1 substitutions per nucleotide position.

**Figure 4:**
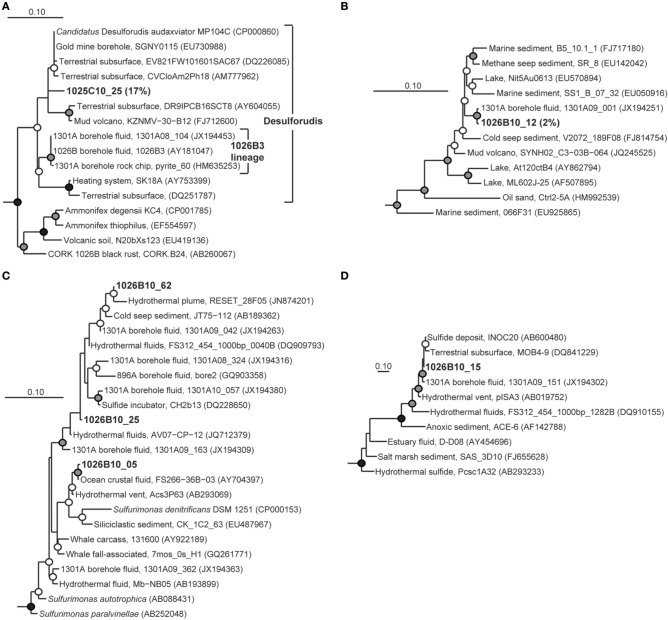
**Phylogenetic relationships of SSU rRNA gene clones from Holes 1025C and 1026B fluids within the phylum *Firmicutes*, related to (A) *Candidatus* Desulforudis audaxviator, (B) RF3, (C) *Sulfurimonas* of the bacterial phylum *Epsilonproteobacteria*, and (D) DHVEG-6 of the domain *Archaea*.** Short length gene clone pyrite_60 was added to the *Candidatus* Desulforudis audaxviator tree after tree construction and bootstrapping and is indicated by a dashed line. Other information as in Figure 3.

Members of the genus *Desulfobulbus*, a group of cultivated *Deltaproteobacteria* that are able to grow by dissimilatory sulfate reduction (e.g., Sass et al., 2002; Suzuki et al., 2007), dominated the clones recovered from Hole 1025C fluid (56% of clones; Table 2). Two monophyletic lineages were detected; one was nearly identical (>99% similarity) to environmental gene clones recovered from Hole 1026B fluids in this study, while the other was most closely related to environmental gene clones from terrestrial and mangrove soil (e.g., Berlendis et al., 2010; Figure 3). The second most abundant group of environmental gene clones from Hole 1025C fluid was related to *Candidatus* Desulforudis audaxviator (17% of clones; Table 2), an uncultivated lineage within the phylum *Firmicutes* that has been detected previously in marine subsurface borehole fluids (e.g., Cowen et al., 2003; Jungbluth et al., 2013) and the terrestrial subsurface (e.g., Moser et al., 2005; Chivian et al., 2008). Interestingly, the environmental gene clones recovered from 1025C form a monophyletic lineage that is distinct from those previously recovered from Holes 1026B and U1301A fluids (Figure 4A). Several other less abundant bacterial lineages were recovered from Hole 1025C, and accounted for 27% of the microbial community (Table 2; Figure 3). No Archaea were recovered from 1025C.

### Hole 1026B fluid community structure

**Figure 5:**
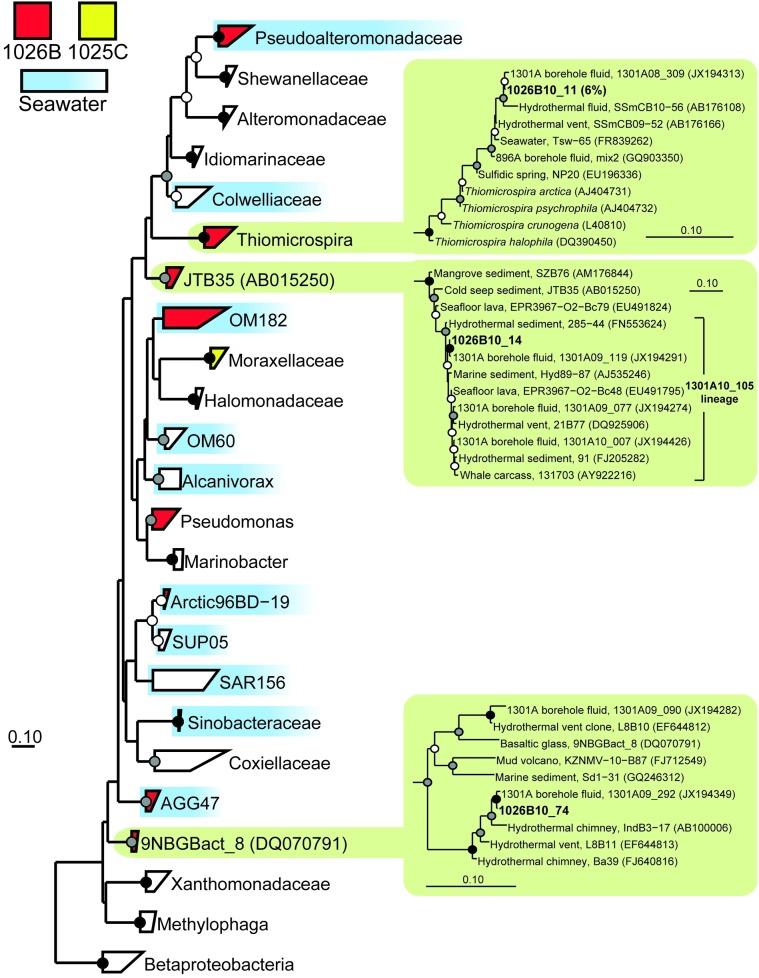
**Phylogenetic relationships of SSU rRNA gene clones related to the phylum *Gammaproteobacteria*, colored as in Figure 3.** A variety of *Betaproteobacteria* were used as outgroups (not shown). Detailed phylogenies are shown for selected lineages. Other information as in Figure 3.

The two most abundant environmental gene clone groups recovered from Hole 1026B fluid were most closely related to the seawater-associated lineages SAR11 (17% of clones) and the *Pseudoalteromonadaceae* (9% of clones), including SAR11 and *Pseudoalteromonadaceae* gene clones recovered previously from Hole U1301A fluids and surrounding bottom seawater (Table 2) (Jungbluth et al., 2013). However, several lineages were recovered from 1026B that, based on the physiology of the most closely related cultivated strains, may rely on inorganic sulfur-containing compounds for growth. These include *Desulfobulbus* (8% of clones) and *Desulfobacula* (6% of clones) of the *Deltaproteobacteria* and *Thiomicrospira* of the *Gammaproteobacteria* (6% of clones) (e.g., Jannasch et al., 1985; Kuever et al., 2001; Pagani et al., 2011) (Table 2). These lineages are also closely related to environmental gene clones from the hydrothermally-influenced marine subsurface (Figures 3, 5) (e.g., Brazelton et al., 2010; Nigro et al., 2012; Jungbluth et al., 2013). Several other low-abundance environmental gene clone lineages recovered from Hole 1026B fluid were most closely related to cultured microorganisms or environmental gene clones that originated from the marine subsurface (Table 2), and accounted for 17% of the microbial community.

### Overlap with previous oceanic crustal fluid studies

Several gene clones recovered from the two CORK fluid samples were closely related to environmental gene clones previously characterized from oceanic ridge flank crustal fluids. Hole 1025C fluids contained gene clones that were highly related to those recovered from Hole 1026B fluids in this study (*Desulfobulbus*; Figure 3), Hole U1301A fluids sampled across multiple years (*Desulfocapsa*; Figure 3) (Jungbluth et al., 2013), and crustal fluids from Baby Bare seamount (*Alkaliphilus*) (Huber et al., 2006). These lineages contain cultured representatives known to be involved in sulfur cycling (Finster et al., 1998; Takai et al., 2001). A lineage of gene clones recovered from Hole 1026B fluids here was discovered to form a monophyletic clade that has been previously detected from fluids (Cowen et al., 2003) and a rusty biofilm (Nakagawa et al., 2006) from CORK 1026B (Figure 3); closest cultivated relatives within the family *Desulfohalobiaceae* of the phylum *Deltaproteobacteria* have also been implicated in sulfur cycling (Zhilina et al., 1997; Sorokin et al., 2008).

Several clones recovered from Hole 1026B had close phylogenetic relation to Hole U1301A fluid clones collected during previous years (Jungbluth et al., 2013), including *Gammaproteobacteria* lineages *Thiomicrospira*, JTB35, 9NGBBact_8 (Figure 5); *Firmicutes* lineage RF3 (Figure 4B); *Epsilonproteobacteria* lineage *Sulfurimonas* (Figure 4C); *Planctomycetes* lineage OM190; *Fusibacter*; and archaeal lineage DHVEG-6 (Figure 4D).

Clustered at 97% similarity, two OTUs were common to Hole 1025C, 1026B, and U1301A fluids (SAR11 and *Desulfocapsa*), while an OTU within the genus *Desulfobulbus* was shared between Holes 1025C and 1026B crustal fluids to the exclusion of U1301A. Excluding OTUs that were also detected in bottom seawater, the Hole 1026B fluid sample characterized here contained a single lineage in common with Hole 1026B fluid samples characterized by Cowen et al. (2003) (*Desulfohalobiaceae)* or Huber et al. (2006) (*Sulfurimonas*).

### Overlap with other marine subsurface habitats

**Figure 6:**
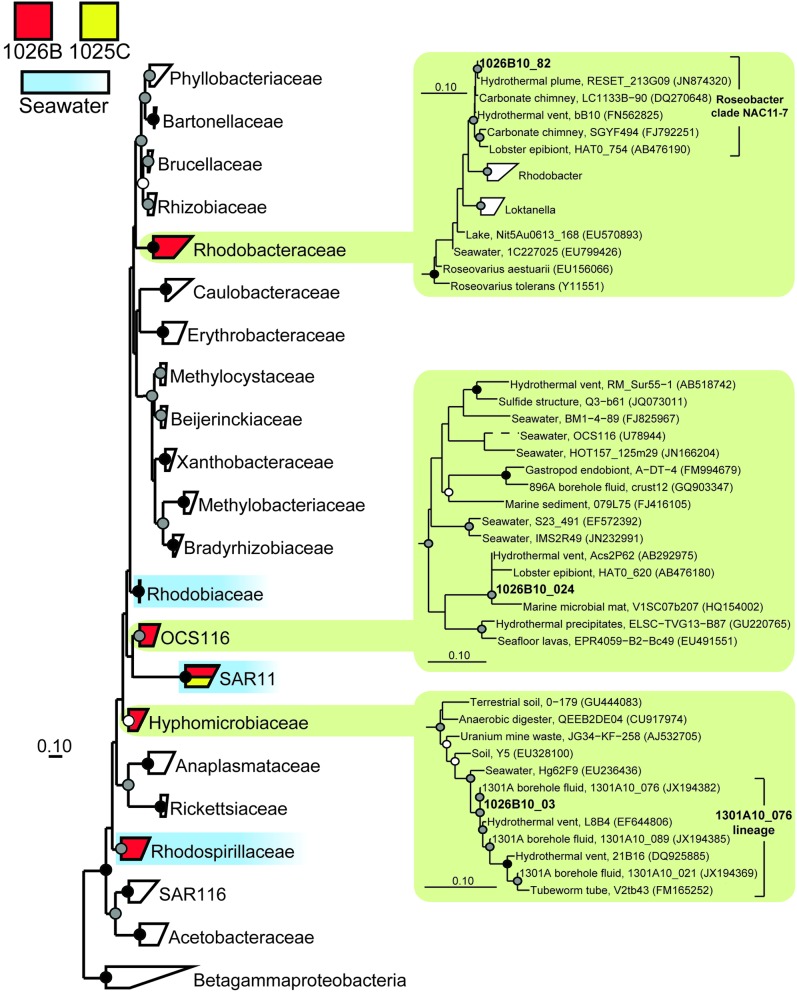
**Phylogenetic relationships of SSU rRNA gene clones related to the phylum *Alphaproteobacteria*, colored as in Figure 3.** Detailed phylogenies are shown for selected lineages. Short length gene clone OCS116 was added after tree construction and bootstrapping and is indicated by a dashed line. Other information as in Figure 3.

In addition to the clones described above, six gene clone lineages from 1025C and 1026B were closely related to sequences previously recovered from marine subsurface habitats typically associated with hydrothermal activity. A group of gene clones related to the mesophilic, sulfate-reducing group *Desulfovibrionaceae* (e.g., Abildgaard et al., 2006; Thabet et al., 2007) was detected in Hole 1025C fluids (Figure 3). Two groups within the *Alphaproteobacteria* were detected that fell within phylogenetic clades predominantly derived from seawater environments (OCS116 and *Rhodobacteraceae*; Suzuki et al., 1997; Wagner-Döbler and Biebl, 2006; Morris et al., 2012); however, phylogenetic inference that included gene clones detected within Hole 1026B fluids revealed that these clades may contain lineages specific to hydrothermally-active subsurface environments (Figure 6). Two additional lineages from 1025C that were related to environmental gene clones previously described from hydrothermal chimneys were detected in marine subsurface fluids for the first time, including *Bacteroidetes* lineage SB-1 and an uncultivated *Firmicutes* lineage related to known *Desulfotomaculum* discovered at Lost City carbonate vents (e.g., Brazelton et al., 2010). However, this lineage was distinct from terrestrial (e.g., Moser et al., 2005) and marine (Cowen et al., 2003; Nakagawa et al., 2006) environmental gene clones previously described from this group. A single gene clone related to environmental gene clones isolated from hydrothermal vents (Kato et al., 2009) and sediments (Davis et al., 2009) within the iron-oxidizing phylum *Zetaproteobacteria* (Emerson et al., 2007; Singer et al., 2011) was also detected in Hole 1026B fluids.

## Discussion

While the CORKs affixed to Holes 1026B and 1025C were not designed with the intent to be exemplar platforms through which to investigate the microbiology of deep subsurface crustal fluids, they nonetheless offer independent points of access that can serve to complement and verify observations made from other CORK platforms that were designed specifically to maintain the biological integrity of retrieved borehole fluids, such as U1362A and U1362B on the JdFR flank (Fisher et al., 2012). As priority for geochemical and microbiological sampling of older boreholes diminishes due to the antiquation of early-generation CORK materials and suboptimal fluid delivery systems, the likelihood of prioritizing research and thus submersible dive objectives in an effort to obtain fluid samples from them also continues to decrease. However, this study add to previous research (e.g., Cowen et al., 2003; Nigro et al., 2012) that demonstrates that early generation CORKs are capable of yielding useful crustal fluid samples for comparative analysis, particularly when significant flushing measures are employed.

The biogeochemical composition of the Hole 1025C fluid sample investigated here was more similar to predicted end-member deep subsurface crustal fluid values than that of the Hole 1026B fluid sample. Efforts taken to minimize the sampling of contaminating seawater were predominantly successful at 1025C, as indicated by depleted O_2_ concentration during sampling and other fluid chemistry suggesting only ~16% bottom water composition, and reflected in low detection of seawater environmental gene clones in the clone library (~10%). In contrast, the 1026B sample yielded at least 50% of typical seawater related environmental gene clones, in agreement with the near-background O_2_ concentration of the collected sample, and further supported by the seawater-like chemical composition of the fluid sample. Unfortunately, it is not possible to estimate if the microbial community followed a conservative mixing model of two end-member fluids consisting of bottom seawater and crustal fluid because fluids collected from both 1026B and 1025C contained a high particulate matter load that prohibited the enumeration of microbial cells via microscopy. It is also not feasible to pinpoint the exact mechanism by which significantly more seawater was introduced into the sample from 1026B as opposed to 1025C; the two boreholes possess vast differences in a variety of factors that includes both CORK characteristics and host environment. However, one potential explanation lies in the observation that the seal of the CORK affixed to Hole 1026B has lost integrity and allows fluid to circumvent the fluid delivery lines of the CORK itself (Fisher et al., 2011). Consistent with this explanation is that gastight samples collected in 2008 were comprised of 99% end-member basement fluids (Lin et al., 2012), but only comprised 3–10% of the gastight sample collected in 2010. In addition, the low pH, extremely high iron concentration of the 2010 1026B sample, and ratios of dissolved Fe(II), total dissolved Fe, and total Fe also indicate significant iron corrosion has taken place within fluid delivery lines and borehole casing.

Concurrent to sampling from Holes 1025C and 1026B, fluid samples were also collected from Hole U1301A for biogeochemical (Lin et al., 2012) and microbial analysis (Jungbluth et al., 2013). Hole U1301A was drilled in 2004 in close proximity to Hole 1026B and affixed with a CORK-II observatory possessing stainless steel fluid delivery lines (Expedition 301 Scientists, 2005), providing an independent access point to 3.5 million years old (Ma) crust and a means to support sample quality assessment from Hole 1026B. The physical proximity of the two boreholes suggests a similar fluid alteration history (Wheat et al., 2010). Despite evidence from O_2_, iron concentrations, and other parameters that the 1026B CORK seal has lost integrity and is allowing seawater intrusion, several environmental gene clone lineages recovered from this sample were closely related to environmental gene clones previously characterized from Hole 1026B fluids collected nearly 12 years ago (Cowen et al., 2003) as well as from 3 successive years (2008–2010) of Hole U1301A fluids characterized previously (Jungbluth et al., 2013). Thus, although contaminated with microorganisms of likely seawater origin, the Hole 1026B sample analyzed here provides independent support for the presence of certain microbial lineages in deep subsurface crustal fluids.

Despite being of higher integrity with regard to seawater intrusion, the microbial community within the Hole 1025C sample analyzed here overlapped little with either the JdFR flank fluid communities characterized previously or the Hole 1026B sample analyzed as part of this study. Two plausible explanations are that the old style CORK system in place at this borehole exerts and influence on the structure of the microbial community, or that the borehole taps an environment that hosts a distinct autochthonous microbial community. While neither scenario can be excluded, it is worth pointing out that the chemical characteristics of 1025C-derived crustal fluids are unique compared to that of the other boreholes investigated here in that they fall between those of bottom seawater and Hole U1301A, which is consistent with its classification within a Hydrothermal Transition Zone (Shipboard Scientific Party, 1997).

When considering the combined dataset from the two new fluid samples analyzed here, lineages within the bacterial phyla *Deltaproteobacteria* (*Desulfocapsa*, *Desulfohalobiaceae*), *Gammaproteobacteria* (JTB35, *Thiomicrospira*, 9NBGBact_8), *Alphaproteobacteria* (*Hyphomicrobiaceae*), *Epsilonproteobacteria* (*Sulfurimonas*), *Firmicutes* (Desulforudis, RF3), *Planctomycetes* (OM190), *Fusibacter*, and one within the archaeal domain (DHVEG-6) have all been previously found in JdFR flank crustal fluid samples (Cowen et al., 2003; Huber et al., 2006; Jungbluth et al., 2013). In the deep terrestrial subsurface, the Desulforudis lineage can make up an extremely high proportion of microorganisms *in situ*, and has been associated with the potential for sulfate reduction, inorganic carbon fixation, and nitrogen fixation via genomic analysis (Chivian et al., 2008). In the marine deep subsurface, this lineage has now been recovered in relatively high proportion in fluids retrieved from 1025C (17%), 1026B (39%; Cowen et al., 2003), and U1301A (36% of 2008 sample; Jungbluth et al., 2013). Interestingly, Desulforudis-related gene sequences from Hole 1025C fluids form a monophyletic lineage with sequences detected from the terrestrial subsurface rather than other marine lineages recovered previously. Thus, within this lineage it appears that there may have been multiple transitions between the terrestrial and marine deep subsurface environments. The functional and evolutionary characteristics that are shared amongst the different deep subsurface Desulforudis-related lineages, and what differences may account for the pattern of evolutionary descent observed here, remain to be discovered.

The recovery of multiple shared lineages provides evidence linking microbial communities from several boreholes along the JdFR flank. This is not altogether surprising, as the CORK-fitted boreholes ostensibly tap in to basalt-hosted deep subsurface crustal fluids in relatively close proximity to one another and experience broad similarities in physical and chemical conditions. While these linkages provide an initial framework for investigating the genetic and evolutionary characteristics of microbial populations in the marine deep subsurface, it would benefit greatly from a genomic approach that extends beyond a relatively conserved, single-gene phylogenetic marker such as the 16S rRNA. In addition, environmental genomics approaches—whether based on single cell whole genome amplification and sequencing or metagenomics—would also help to illuminate the functional attributes of these lineages, as nearly all contain no close phylogenetic relatives in laboratory culture.

A subset of clones that bear reasonably close phylogenetic relationships to known isolates allow for some speculation regarding the functional characteristics of a few microbial lineages detected in Holes 1025C and 1026B. A common theme of the *Deltaproteobacteria* lineages related to clones described in this study is the ability to utilize low-molecular weight organic compounds (e.g., acetate, formate, pyruvate) and intermediate redox-state sulfur compounds as electron donors (e.g., Suzuki et al., 1997; Abildgaard et al., 2006); in some cases hydrogen can be used directly (e.g., Kuever et al., 2001; Alazard et al., 2003). Clone lineages from Hole 1025C and Hole 1026B fluids related to anaerobic, heterotropic *Deltaproteobacteria* groups (e.g., *Desulfobulbus*, *Desulfovibrio*) were in relatively high abundance, while clones related to potentially autotrophic lineages of *Gammaproteobacteria* that perform the oxidation of intermediate sulfur compounds (e.g., *Sulfurimonas*, *Thiomicrospira*, and Arctic96BD-19; Jannasch et al., 1985; Inagaki et al., 2003; Marshall and Morris, 2013), were also present.

Microbial lineages related to mesophiles that perform anaerobic sulfur-cycling processes such as the disproportion and/or reduction of sulfate (e.g., *Desulfobulbus, Desulfovibrio*, *Desulfocapsa*) were detected in high abundance from Hole 1025C. However, lineages related anaerobic mesophiles were also members of the Hole 1026B fluid sample, which is inconsistent with the expected borehole fluid temperature (~64°C) within Hole 1026B. It is possible that the suspected seawater intrusion in this location has cooled the immediate surroundings of the permeated basement aquifer and is selecting for a mesophilic microbial community. Consistent with this idea is the notable absence of relatives of the thermophilic sulfate-reducing lineage *Archaeoglobus*, which is a lineage that has been consistently detected from borehole 1026B in previous studies (Cowen et al., 2003; Nakagawa et al., 2006; Steinsbu et al., 2010). Microbial lineages related to known methanogens were absent, and only a single gene clone related to a known iron oxidizer was recovered, from Hole 1026B (i.e., *Zetaproteobacteria*). This suggests that either iron oxidation is not a common metabolic trait, or is being performed by as-yet unidentified iron oxidizers in this environment.

In summary, the basement fluid samples investigated here leverage the spatial array of borehole observatories located on the JdFR flank to reveal some aspects of deep subseafloor microbial community biogeography, particularly with regard to the overlap in microbial community members between boreholes located in close proximity, 1026B and 1301A. While 1026B and 1025C are not ideal targets for assessing the quantitative characteristics of microorganisms residing within the deep subseafloor, here they yield important, independent evidence for the presence of a number of phylogenetic lineages within the basalt-hosted deep subseafloor biosphere.

### Conflict of interest statement

The authors declare that the research was conducted in the absence of any commercial or financial relationships that could be construed as a potential conflict of interest.
